# Deep Sequencing of MHC-Adapted Viral Lines Reveals Complex Recombinational Exchanges With Endogenous Retroviruses Leading to High-Frequency Variants

**DOI:** 10.3389/fgene.2021.716623

**Published:** 2021-08-27

**Authors:** Earl A. Middlebrook, Derek L. Stark, Douglas H. Cornwall, Jason L. Kubinak, Wayne K. Potts

**Affiliations:** ^1^School of Biological Sciences, University of Utah, Salt Lake City, UT, United States; ^2^Biosecurity and Public Health, Los Alamos National Laboratory, Los Alamos, NM, United States; ^3^Department of Pathology, University of Utah, Salt Lake City, UT, United States; ^4^Department of Pathology, Microbiology and Immunology, University of South Carolina School of Medicine, Columbia, SC, United States

**Keywords:** recombination, ERV, host adaptation, serial passage, Friend virus complex, murine leukemia virus, experimental evolution

## Abstract

Experimental evolution (serial passage) of Friend virus complex (FVC) in mice demonstrates phenotypic adaptation to specific host major histocompatibility complex (MHC) genotypes. These evolved viral lines show increased fitness and virulence in their host-genotype-of-passage, but display fitness and virulence tradeoffs when infecting unfamiliar host MHC genotypes. Here, we deep sequence these viral lines in an attempt to discover the genetic basis of FVC adaptation. The principal prediction for genotype-specific adaptation is that unique mutations would rise to high frequency in viral lines adapted to each host MHC genotype. This prediction was not supported by our sequencing data as most observed high-frequency variants were present in each of our independently evolved viral lines. However, using a multi-variate approach to measure divergence between viral populations, we show that populations of replicate evolved viral lines from the same MHC congenic mouse strain were more similar to one another than to lines derived from different MHC congenic mouse strains, suggesting that MHC genotype does predictably act on viral evolution in our model. Sequence analysis also revealed rampant recombination with endogenous murine leukemia virus sequences (EnMuLVs) that are encoded within the BALB/c mouse genome. The highest frequency variants in all six lines contained a 12 bp insertion from a recombinant EnMuLV source, suggesting such recombinants were either being favored by selection or were contained in a recombinational hotspot. Interestingly, they did not reach fixation, as if they are low fitness. The amount of background mutations linked to FVC/EnMuLV variable sites indicated that FVC/EnMuLV recombinants had not reached mutation selection equilibrium and thus, that EnMuLV sequences are likely continuously introgressing into the replicating viral population. These discoveries raise the question: is the expression of EnMuLV sequences in mouse splenocytes that permit recombination with exogenous FVC a pathogen or host adaptation?

## Introduction

Experimental evolution is a powerful tool for understanding adaptation of organisms to their environments, such as the adaptive response of a pathogen to its host environment. Serial passage, whereby a pathogen is successively transmitted between a series of hosts, is a potent experimental evolutionary technique for generating rapid adaptive responses by a pathogen. Experiments have demonstrated that serial passage generally leads to increased pathogen fitness and virulence in hosts-of-passage, while fitness and virulence tend to decrease in alternative host genotypes (Ebert, 1998; Kubinak et al., 2012). These decreases are presumably due to host genotype-specific adaptations by the pathogen that are maladaptive in the context of other host genotypes or species, otherwise known as antagonistic pleiotropy or adaptive tradeoffs (Gandon, 2004; White et al., 2020).

Antagonistic coevolution between host and pathogen has been experimentally shown to increase the rate of molecular evolution (bacteria/phage, Paterson et al., 2010) and increase host genetic variation in the following systems: beetle/microsporidian (Bérénos et al., 2011), daphnia/multi-parasite (Wolinska and Spaak, 2009), and snail/trematode (Dybdahl and Lively, 1998). Increased host genetic variation is often thought to be caused by negative frequency-dependent selection (NFDS), whereby pathogens adapt to common host alleles, thereby giving uncommon alleles a selective advantage (Haldane, 1949; Hamilton, 1993). MHC molecules are cell-surface glycoproteins that bind and present peptide antigens on the cell surface. MHC:peptide complexes serve as agonist ligands for T cell receptors and thus play a fundamental role in adaptive immunity. In most vertebrate populations surveyed, the genes encoding MHC molecules are exceptionally polymorphic (e.g., >17,000 HLA class I alleles in humans) (Robinson et al., 2020). Recently, NFDS has been demonstrated for specific MHC alleles in guppy and stickleback fish (Bolnick and Stutz, 2017; Phillips et al., 2018).

**FIGURE 1:**
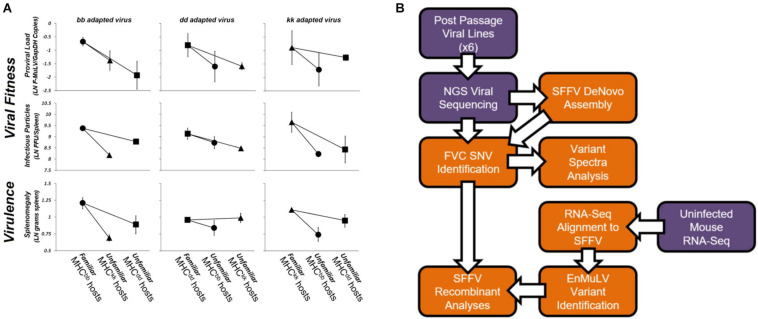
Friend virus complex adapted to a specific MHC genotype and study workflow. Passaged FVC shows strong fitness and virulence tradeoffs when infecting unfamiliar (novel) host genotypes **(A)**. Symbols reflect host genotypes (circle = MHC^bb^ hosts, square = MHC^dd^ hosts, triangle = MHC^kk^ hosts) and represent the log transformed least square (LS) means of two replicate tests (error bars represent the range of means for each test). Comparisons of patterns of pathogen fitness and virulence between virus stocks (denoted bb passaged virus, dd passaged virus, and kk passaged virus) across the three host genotypes shows that pathogen fitness and virulence is host genotype-specific with tradeoffs in the ability of virus adapted to one MHC to productively infect and cause virulent disease in other (unfamiliar) host MHC genotypes. Viral fitness (12 of 12 comparisons) and disease virulence (5 of 6 comparisons) are higher when a passaged virus stock is exposed to a familiar (i.e., its host genotype-of-passage) versus an unfamiliar MHC genotype (i.e., a genotype the virus hasn’t been passaged through). Data are shown on a natural-log scale. Adapted from Kubinak et al. (2012). The bioinformatics workflow used in the present study is diagramed in **(B)**. Sample or data inputs are depicted in purple while computational steps are shown in orange. Arrows show workflow order.

Using serial passage, Kubinak et al. (2012) demonstrated that Friend virus complex (FVC) adapts to specific host MHC genotypes, resulting in trade-offs in viral fitness and virulence when adapted viruses are exposed to unfamiliar host MHC genotypes. Figure 1 shows data from this study where FVC was adapted to three MHC congenic BALB/c mouse strains that possess unique sets of MHC alleles. After 10 rounds of 12-day passages (i.e., 120 days in each host genotype), adapted viral lines were tested for host-genotype-specific adaptation by infecting cohorts of mice from each of the three homozygous MHC genotypes (denoted as bb, dd, and kk). In 17 of 18 comparisons, viral fitness or virulence was greater in the host-genotype-of-passage (i.e., “familiar”) than in “unfamiliar” host genotypes (Figure 1). The observed trade-offs lead to fitness advantages for hosts possessing novel (i.e., unfamiliar to pathogen) MHC alleles and supports NFDS operating on MHC loci, potentially contributing to its extreme allelic diversity. A similar pattern was demonstrated when FVC was allowed to adapt to mouse strains that differed across the entire genome (Kubinak et al., 2013). Likewise, population level MHC diversity has been shown to reduce pathogen fitness and virulence evolution (Cornwall et al., 2018). To date, these three studies provide the only experimental data to support that adaptive tradeoffs of pathogens can drive NFDS on MHC alleles in mammals. Thus, studies in mammals and fish now indicate that NFDS can have major consequences for both host and pathogen evolution.

Long co-evolutionary histories between vertebrate hosts and retroviruses have led to the accumulation of endogenous retroviral sequences (ERVs) within host genomes. These ancient sequences, derived from germ-line insertions of retroviral genomes, account for 8 and 10% of the human and mouse genomes, respectively (Belshaw et al., 2004; Kozak, 2015). Once thought to be genomic parasites or neutral graveyards, it is becoming clear that ERVs can span the parasite/mutualist continuum (Dewannieux and Heidmann, 2013; Hurst and Magiorkinis, 2015; Malfavon-Borja and Feschotte, 2015). Laboratory mice, *Mus musculus*, have many copies of murine leukemia viruses (MuLVs) integrated into their genomes and can also be susceptible to their exogenous viral counterparts (Kozak, 2013). One such virus, FVC, has become a powerful tool for investigations of leukemia pathogenesis, hematopoiesis, and anti-retroviral immunology (Friend, 1957; Hasenkrug and Chesebro, 1997). FVC consists of two viral genomes: the replication-competent (i.e., helper) Friend Murine Leukemia Virus (F-MuLV), and the replication-defective Spleen Focus Forming Virus (SFFV). When certain strains of mice are infected with the exogenous F-MuLV, recombination with envelope (*env*) genes of endogenous MuLVs (EnMuLVs) can generate viruses that possess novel cell tropisms that are referred to as polytropic-MuLVs (Evans and Cloyd, 1984). SFFV evolved from such an event, and possesses several additional genetic modifications that make it uniquely pathogenic.

In SFFV, a 6 base pair (bp) duplication and a 1 bp insertion at the 3′ end of the recombinant *env* gene causes a frameshift mutation resulting in a premature stop in envelope protein translation and removal of the cleavage site between the envelope transmembrane and surface unit, creating a membrane-bound and truncated *env* glycoprotein (gp55) (Troxler et al., 1978; Chesebro et al., 1990; Watanabe et al., 1991). Critically, Gp55 co-localizes with and constitutively activates the mouse erythropoietin receptor expressed on the surface of infected erythroblasts, causing massive cellular proliferation and gross enlargement of organs associated with erythroblast terminal differentiation (spleen and liver). However, SFFV’s novel envelope function comes at the cost of making functional viral particles, leaving SFFV dependent on complementation by F-MuLV’s functional envelope protein (Steeves and Lilly, 1977). Gp55-induced cell proliferation increases viral fitness in two ways: first by leading to replication of F-MuLV and SFFV proviruses in proliferating cells, and second by increasing the number of target cells able to be super-infected by F-MuLV. Thus, SFFV and F-MuLV work in concert to cause the severe erythro-proliferative disease observed in susceptible mice (reviewed in Myers and Hasenkrug, 2009).

Here we report the results of ultra-deep sequencing of FVC viral lines previously adapted to three MHC congenic BALB/c mouse strains (Figure 1; Kubinak et al., 2012) with the objective of characterizing the genetic basis of MHC-specific adaptation. The central prediction to be tested in our study was that viral variants would rise to high frequency due to selective sweeps, and that these variants would be unique to viral lines adapted to different MHC genotypes. Using this next generation sequencing data (Figure 1B), we found little evidence in support of this prediction. Instead, we found a high number of low frequency variants, possibly contributing to host genotype-specific adaptation through cryptic mechanisms. Strikingly, our deep sequencing efforts also revealed repeated recombination between EnMuLVs present in the mouse genome and exogenous SFFV. One section of these common recombinant EnMuLV elements repeatedly rose to high frequency in all of the evolved viral lines indicating that MHC-genotype-independent genetic exchange between SFFV and endogenous EnMuLV sequences is a major feature of FVC evolution during serial passage through BALB/c mice.

## Results

### Unpassaged Virus Sequencing

To discover the genetic basis of observed increases in fitness and virulence of FVC, we first sequenced FVC provirus from a cell culture stock of virus used to create the serial passage strains used in this study, hereafter referred to as FVC-Boiclone (Kubinak et al., 2012). The viral stock was derived from an NIH 3T3 cell line containing integrated genomes of F-MuLV (NC_001362.1: strain FB29) and SFFV (SFFV-AP-L strain), described in Wolff et al. (1985). Although the viral genomes present in FVC-BC have been published, we sequenced the pre-passage viral genomes (referred to in this manuscript as F-MuLV-BC and SFFV-BC) to ensure that there had been no significant changes to the viral sequences during cell culture maintenance. Relative to the published sequence, F-MuLV-BC has five mutations at less than 0.03 allele frequency and six with frequencies between 0.17 and 0.26. Of the six high frequency variants, one is synonymous, four are non-synonymous, and one is a nonsense mutation. The frequencies of all detected sequence variants, with one exception, approached zero after the first two rounds of passage *in vivo* (Supplementary Figure 2). This result suggests that these mutations arose due to drift or cell culture adaptation, and that they are purged by purifying selection during passage. Alignment of SFFV reads to the reference sequence revealed many fixed single nucleotide variants (SNVs) along with several indels, indicating that the SFFV sequence in the inocula had acquired many changes relative to the SFFV-AP-L reference (Wolff et al., 1985). These changes likely arose during expansion of the original cell culture stock within our lab.

To accurately reconstruct the sequence of SFFV in the inocula (SFFV-BC), sequencing reads were *de novo* assembled. The longest sequence generated by SPAdes was a 11 kb contig. Upon alignment of the expected SFFV-AP-L sequence with this contig, it became clear the assembled contig was a tandem repeat of an SFFV related sequence of about 4.8 kb. This was corroborated by aligning the contig against NCBI’s non-redundant nucleotide database. Illumina reads were aligned back to a single unit of this tandem repeat, which represented a full SFFV genome (*gag* and *env* genes flanked by 5′ and 3′ LTRs). No structural variants and few SNVs, all of low frequency, were detected. This supports the generated sequence as the inocula SFFV genome. Furthermore, this genome is recoverable after ten rounds of passage in mice, indicating that it is packaged into virions and is infectious (see Passaged Virus below). Interestingly, there are no sequences in NCBI’s non-redundant nucleotide database that resemble the inferred SFFV-BC genome across its whole length, indicating this is a novel SFFV-like viral sequence (Supplementary Figure 2). This novel SFFV sequence is available with accession MZ614724 from NCBI’s nucleotide database.

### Passaged Virus Sequencing

**FIGURE 2:**
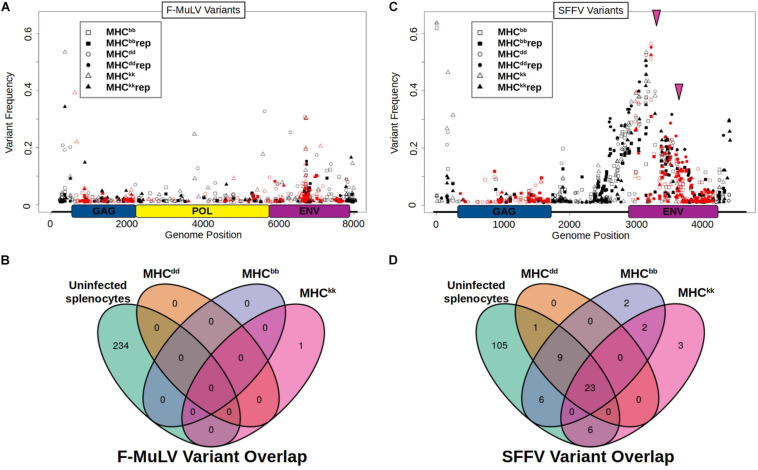
F-MuLV and SFFV variants detected in post passage viral lines. **(A,B)** SNVs identified in **(A)** F-MuLV and **(B)** SFFV are shown. Host MHC genotypes are indicated by data point symbol. Independent biological replicates are denoted by filled or open symbols. Non-coding or synonymous mutations are shown in black while non-synonymous mutations are shown in red. Pink Arrows indicate genomic positions of common, in-frame insertions of post passage virus populations. Genomic maps of viral open reading frames are shown on the *x* axis of plots. GAG, group antigens; POL, polymerase and ENV, envelope. **(C,D)** Overlap between high frequency (>10%) variants from **(C)** F-MuLV and **(D)** SFFV populations and ERVs from uninfected mouse RNA-seq are shown using Venn-diagrams. SNVs detected in both replicates of each MHC host genotype are compared to identify MHC specific variants and those shared between different host MHC types. MHC genotype of passage is shown next to ovals. Variants detected in RNA seq of uninfected BALB/c splenocytes are shown in green oval. Variants below a population frequency of 10% are not shown.

In an effort to identify viral variants associated with genotype-specific adaptation to mouse MHC, integrated proviral sequences were amplified from post-passage viral stocks and sequenced on an Illumina MiSeq. Alignment of post-passage next-generation sequencing (NGS) reads to F-MuLV-BC and the *de novo* assembled SFFV-BC reference sequence showed there were no fixed variants after ten serial passage rounds in any of the six post-passage viral stocks generated. The maximum variant frequency identified was 65%. The majority of F-MuLV post-passage populations had > 50 variants detected across their entire sequence, ranging in frequency from 1 to 10% and with 1–4 per passage line that are greater than 20% (Figure 2A). However, there was only one sequence variant at a frequency > 10% that was shared between biological replicates and also unique to a specific host MHC genotype (Figure 2B, MHC^kk^), indicating that adaptation of F-MuLV during serial exposure to the same MHC genotype does not result in predictable patterns of SNV evolution or selective sweeps of unique variants. To test the prediction that F-MuLV would escape host immune pressure by mutating T-cell epitopes, we analyzed the overlap between SNVs and either predicted or empirically identified T-cell epitopes. In agreement with the above results, we did not detect enrichment for mutations within expected immune epitopes (Supplementary Table 2). These data together indicate that host specific phenotypic adaptation does not have a signature consistent with selective sweeps or viral escape from T-cell responses.

In stark contrast to post-passage F-MuLV, SFFV sequencing reveals many genomic changes in response to serial passage in mice (Figure 2C). Although no SFFV lines have fixed variants, all six post-passage viral populations show many segregating SNVs around genomic position 3170 with a breadth spanning approximately from position 2400 to 3900. The majority, 34 of 52, of the high frequency variants are shared among virus lines from at least two different host genotypes and 23 of 52 are shared among all six post-passage viral lines (Figure 2D). Additionally, there are two high-frequency insertions in post-passage SFFV that are shared across all six post-passage virus populations: a 12 bp insertion of high frequency (25–52%) at the 5′ end of the *env* gene, position 3176 and a 3 bp insertion 241 bp downstream of that (Figure 2C, pink arrows). Interestingly, the 12 bp insertion converts the SFFV-BC envelope sequence to that of several published SFFV sequences, including the expected high virulence SFFV-AP-L bioclone sequence (Supplementary Figure 3), which indicates that the apparent insertion sequence did not originate *de novo* through polymerase error, but instead is likely homologous to these published sequences.

### MHC-Specific Variant Spectra

**FIGURE 3:**
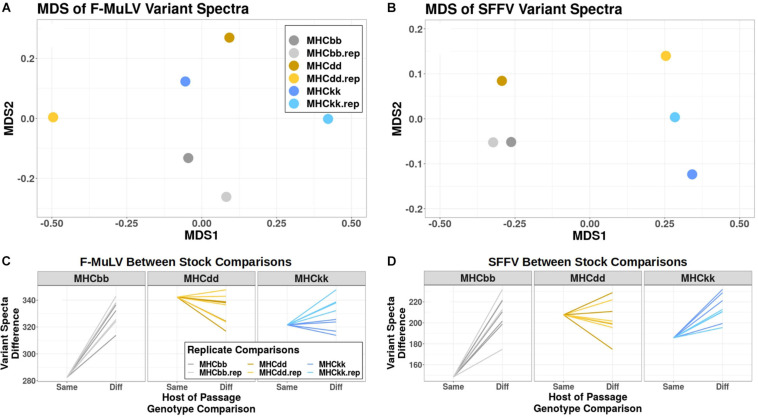
Major histocompatibility complex specific variant spectra. **(A,B)** Multi-dimensional scaling plots of **(A)** F-MuLV and **(B)** SFFV variant spectra are shown. Initial distances were calculated with the Canberra method. Colors represent different host of passage MHC genotypes: Gray-MHC^bb^, yellow-MHC^dd^, and blue-MHC^kk^. Points from biological replicates (independent passage lines from the same host MHC genotype) are shown in lighter colors. **(C,D)** Comparisons of Canberra distances between variant spectra of **(C)** F-MuLV and **(D)** SFFV viral populations are shown, with the same color scheme used as panels **(A,B)**. The *X* axis denotes whether the distance reported is between stocks derived from the “Same” host genotype (i.e., MHC^bb^ and the MHC^bb^ replicate) or between stocks derived from different (“Diff”) host genotypes (i.e., MHC^bb^ and MHC^kk^). bb, dd, and kk show which host genotype is the reference “Same” distance. Positive slopes between “Same” and “Diff” indicate that replicates from the same host environment are more similar than replicates from alternative MHC genotypes.

Because there was little evidence to support high frequency variants being responsible for phenotypic patterns of genotype-specific viral adaptation, we chose to more broadly consider the effect of MHC genotype on the entire viral mutant spectra. To do this, we compared the degree of similarity in mutant spectra that emerge after serial passage through different host genotypes using multi-dimensional scaling (MDS), a data reduction method for determining relationships between samples based on high-dimensional datasets (many SNVs). Figures 3A,B are representative MDS plots illustrating relationships between variant spectra of all six adapted lines for F-MuLV and SFFV variant populations, respectively. To determine if viral lines derived from the same host genotype were statistically more similar to one another than to viral lines derived from other host genotypes, we compared computed Canberra distances used to generate these MDS plots. With respect to F-MuLV mutant spectra, MHC^bb^-derived viral lines demonstrated high similarity (Figure 3A). Specifically, MHC^bb^ replicate lines are statistically more similar to one another (lower distance) than to mutant spectra derived from other MHC genotypes, with positive slopes for all comparisons (single sample *t*-test, *p*-value: 1.8^∗^10^–6^) (Figure 3C). This was not true of MHC^dd^-derived variant populations, and was trending but non-significant for MHC^kk^-derived variant populations (single sample *t*-test, *p*-value: 0.09) (Figure 3C). With respect to SFFV mutant spectra, clustering by host-genotype-of-passage was more clearly observed. Again, MHC^bb^-derived viral lines demonstrated high similarity in SFFV variant spectra compared to alternative host genotypes (single sample *t*-test, *p*-value: 2.56^∗^10^–5^) (Figures 3B,D). Additionally, MHC^kk^-derived viral lines also demonstrated strong patterns of host-specific SFFV variant spectra (single sample *t*-test, *p*-value: 4.6^∗^10^–4^). Again, MHC^dd^-derived lines do not show significant patterns of genotype specific adaptation (Figure 3D). Together, these analyses indicate that MHC^bb^ strongly sculpts the variant spectra of FMuLV and SFFV populations, while MHC^kk^ appears to only sculpt SFFV populations, and MHC^dd^ has little predictable effect on variant spectra of either FVC constituent. These data suggest that the population structure of post-passage variant spectra is, to variable degrees, determined by host MHC genotype.

### Expression of Polytropic MuLVs in Mouse Splenocytes

**FIGURE 4:**
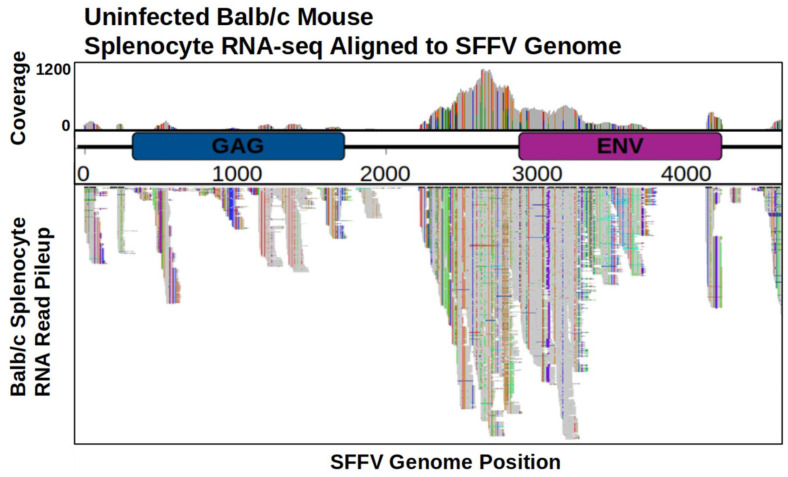
Expression of EnMuLVs from uninfected mouse splenocytes aligned to SFFV-BC reference genome. Position of read alignment is on the *X* axis with a schematic of the annotated SFFV-BC genes. Gray profile above the genome map shows relative coverage of EnMuLV RNA-seq and gray bars below the genome map show actual read alignments. The maximum of EnMuLV read coverage across the SFFV-BC genome is 1112 from a total of 26M initial read pairs. SFFV sites with differences between aligned EnMuLV reads and the SFFV-BC reference sequence are shown as vertical colored bars within the gray coverage profile and aligned reads, with changes noted by color: green-A, blue-C, red-T, gold-G, and purple-Insertions. GAG, group antigen; ENV, envelope gene.

The genomes of many inbred laboratory mouse strains possess EnMuLVs (Kozak, 2015). The presence of common SNVs and insertions between independent post-passage viral populations led to the hypothesis that they arose through recombination with EnMuLV sequences. Because retroviral recombination occurs via strand-switching during reverse transcription of the viral RNA into viral DNA (Lanciault and Champoux, 2006), we sought to determine if BALB/c mice actively transcribed EnMuLV sequences that could serve as template for recombination events. To do this we tested if SFFV-like EnMuLVs were present in RNA-seq data sets generated from uninfected BALB/c splenocytes (SRA accessions ERR216358, ERR216374 ERR216375, ERR216376, ERR216377 from project PRJEB2931). Alignment of these RNA-seq reads to SFFV-BC shows mice express homologous sequences, which likely derive from EnMuLVs (Figure 4). The SFFV-BC region showing highest coverage of EnMuLV expression is the same region which harbors common high-frequency variants detected in post-passage SFFV populations. Furthermore, RNA-seq data of uninfected splenocytes show BALB/c mice express EnMuLV loci that carry the same alleles as the recombinant SFFV-ERV (Supplementary Figure 4). Of the variants above a frequency of 10%, SNVs called from RNA-seq reads that aligned to SFFV-BC accounted for 23/34 SFFV variants shared among multiple host genotypes and 23/23 SNVs shared among all viral populations (Figure 2D). Because EnMuLVs are expressed in SFFV target cells and show the same variant pattern as the post-passage SFFV sequences, it is likely that these EnMuLVs, through recombination, are a contributing source of genetic variation observed in our post-passage SFFV sequences.

**FIGURE 5:**
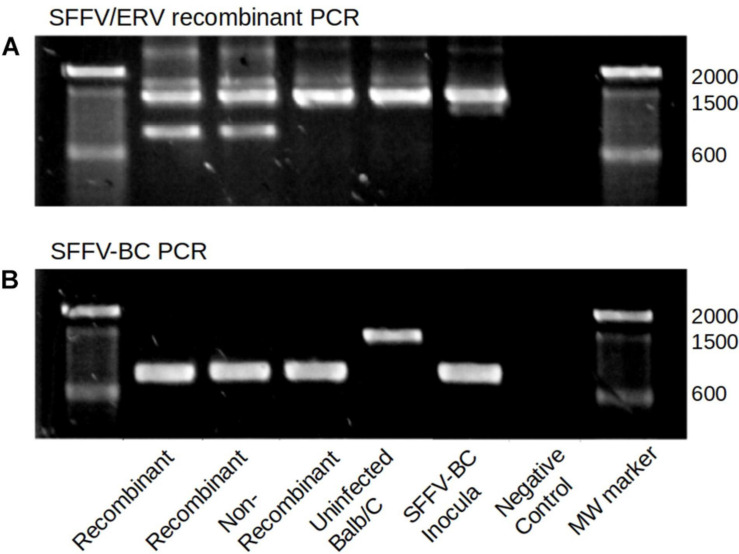
SFFV/ERV recombinant and SFFV-BC specific PCR. **(A)** Top lanes show products of ERV specific forward and SFFV reverse primers. **(B)** The bottom lanes show products of SFFV-BC forward and SFFV-BC reverse primers. The expected product size is 850 for both reactions. Higher molecular weight products are likely non-specific amplification of homologous endogenous MuLV sequences due to using one primer with complementary sequences within the mouse genome. The two representative passage lines which showed presence of SFFV/ERV (Recombinants) in deep sequencing reads, also show the expected SFFV/ERV amplicon band, while all samples negative for recombinant by sequencing do not show this amplicon (Non-recombinant, Uninfected BALBb/c, and SFFV-BC inocula).

To independently confirm SFFV-EnMuLV recombination and rule out common variants detected in post-passage SFFV populations as artifacts of PCR-induced recombination between SFFV and EnMuLVs during NGS library preparation, SFFV-BC and SFFV-ERV recombinant-specific PCR primers were used in PCR reactions of whole-spleen DNA extractions. Forward primers specific for inocula (SFFV-BC) or EnMuLV sequence were used with a downstream reverse primer specific for the SFFV inocula sequence outside of the recombinant area. Figure 5 shows recombinant-specific (Figure 5A) and inocula-specific (Figure 5B) PCR reactions for five different virus populations. Importantly, while two representative samples with inferred recombination by NGS data do show SFFV-EnMuLV recombinant DNA by PCR, DNA from uninfected mice, mouse cells infected with FVC-BC, or an independent passaged virus stock not showing recombination by NGS, show no amplification of the recombinant sequence (Figure 4, 850 bp band). These data illustrate that detection of recombinants by NGS protocols does not represent artifacts resembling SFFV-EnMuLV recombinants in the post-passage viral populations.

### Viral Recombination Landscape

**FIGURE 6:**
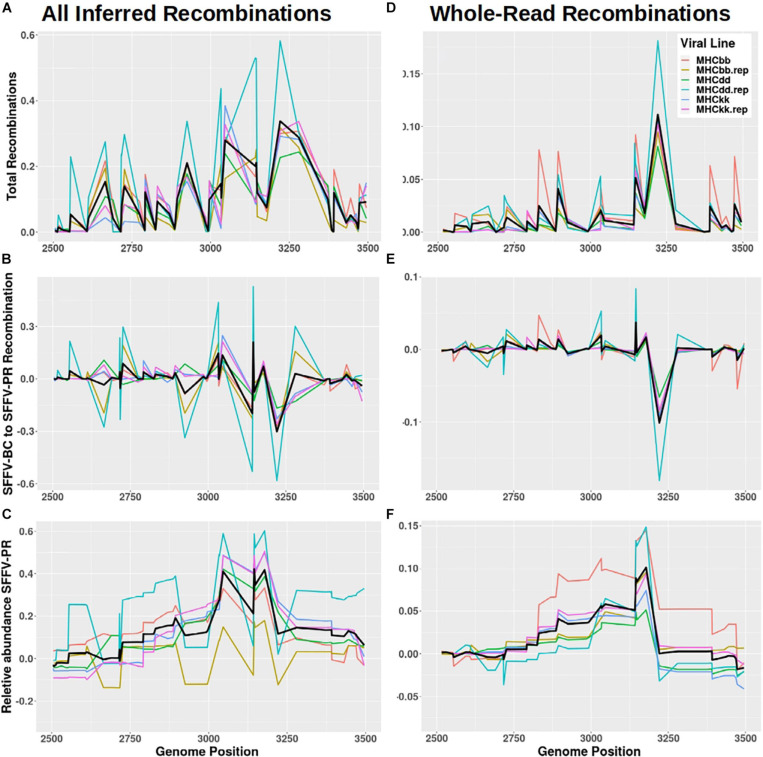
SFFV/ERV recombinants detected in SFFV viral reads. **(A,D)** The total frequency of recombinations, **(B,E)** the frequency of net SFFV-BC to ERV recombinations, and **(C,F)** a rolling sum of net SFFV-BC to ERV recombinations (i.e., the frequency of Polytropic MuLV sequences across the genome inferred by detected recombinations) are all plotted against the SFFV-BC genome. Panels **(A–C)** depict all inferred recombinations while **(D–F)** counts only reads which had a single inferred recombination. Colors denote the six independent passage lines with the average values plotted in black.

To identify recombination hotspots, reads from post-passage SFFV sequencing were compared with the SFFV-BC reference sequence and a representative EnMuLV (chr12:69546811–69 555877 of GRCm39) carrying the majority of ERV-derived SNVs. A custom script was used to assign variants originating from SFFV-BC or EnMuLV haplotypes. Every instance a read showed an SFFV-BC allele adjacent to an EnMuLV allele, a recombination was inferred between the two positions, noting which recombination direction it represented, SFFV-BC to EnMuLV or EnMuLV to SFFV-BC (relative to orientation of the SFFV-BC reference). The plot of all recombinations detected in passaged virus shows that there are hot and cold sections of recombination, with greater positive values indicating common SFFV-BC to EnMuLV or EnMuLV to SFFV-BC recombinations (Figure 6A). Low values indicate that SFFV-BC and EnMuLV haplotypes are conserved within the replicating SFFV population. Figure 6B illustrates that many pairs of alleles across the SFFV genome show directional recombination, with reads becoming more EnMuLV-like with proximity to the variant peak at position 3150. The inferred total number of recombinant SFFV-EnMuLV genomes is shown in Figure 6C. This plot shows a much more complicated recombination landscape than anticipated by simple recombination followed by directional selection or by many instances of EnMuLV sequences introgressing into the SFFV population.

To reduce noise caused by multiple recombinations per Illumina read, sequences with only one recombination across their length are counted. When this is done, the directional recombination leading to the peak at around position 3150 is clearer (Figures 6D–F). Figure 6E shows that, on average, 5′ of the peak the direction of recombination is biased SFFV-BC to EnMuLV and 3′ of the peak recombinations are biased EnMuLV to SFFV-BC. While this approximation based on per-read recombinations captures the shape of the recombinant variant frequencies well (Figure 2B vs. Figure 6F), it fails to capture the magnitude of the variants (∼60% SNVs from sequencing vs. 10% from recombinant analysis). This indicates that many individual genomes within the viral population are the product of multiple recombination events, which have reduced some EnMuLV haplotype lengths to contain one SNV, as indicated above, or that several of the EnMuLV alleles are left out of the analysis because the representative EnMuLV does not carry them.

### Age of Recombinants

**FIGURE 7:**
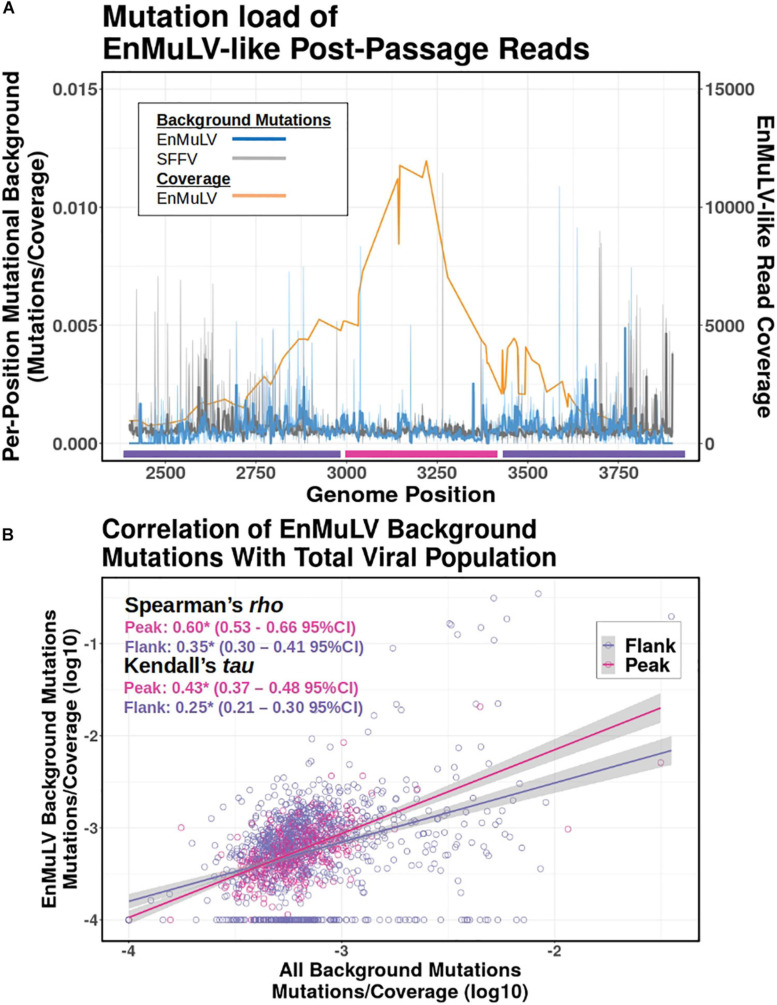
Mutational background of EnMuLV vs. total SFFV population. **(A)** Average fraction of background mutations detected in Illumina reads from 6 post-passage viral populations on EnMuLV-like reads (light blue) and in the total viral population (gray) aligned to the SFFV-BC genome (*x*-axis). Values are calculated as [number of detected variants at position X/estimated coverage at position X]. These raw data are plotted with fine lines while thick lines represent Tukey’s 3R smoothed data to aid visualization. The average coverage of EnMuLV-like reads in the data set are plotted in orange, with scale on the right *y*-axis. Pink and purple lines along the x-axis delineate genome regions “Peak” and “Flank” used in **(B)**. **(B)** The correlation between EnMuLV and Total Mutational Backgrounds for the “Peak” and “Flank” genomic regions are shown. Pink points correspond to genomic positions of SFFV-BC with high EnMuLV-like read coverage (positions 3000–3400) while purple points correspond to the flanking genomic positions (2700–2999 and 3401–3700). Trend lines are inferred from simple linear regressions. Because of non-normality, results for non-parametric Spearman and Kendal correlation tests are shown, with pink and purple corresponding to Peak and Flank genomic regions (respectively) and (*) noting significance of test performed. In parentheses, 95% confidence intervals for tests statistics tau and rho are shown.

Because an ERV reconstituted in a replicating retroviral population will start accruing mutations at the rate of the retrovirus due to replication via retrotranscription, we quantified the background mutational load of EnMuLV-like reads as a proxy for the age of the recombinant sequences. Figure 7 shows average values for data from all 6 post-passage viral populations in this study. The analysis shows that replicating EnMuLV-like sequences in this viral population largely have similar mutational backgrounds relative to the reference-like SFFV sequences (Figure 7A, blue vs. gray lines), indicating that the EnMuLV sequences are close or have reached mutation/selection balance. Under the peak in coverage of EnMuLV-like sequences (orange line), the correlation of EnMuLV and SFFV mutational background appears to be highest. Linear regressions of EnMuLV-like read and SFFV mutational background across the reference genome shows that the correlation is strongest between viral reads that align close to the peak of recombinant frequency (pink) as opposed to the flank (purple) (Figure 7B). Because these data are non-normally distributed, Spearman and Kendall rank correlation tests were used to test for significant relationships between EnMuLV and SFFV mutational backgrounds. Both tests show that EnMuLV and SFFV-BC background mutations are correlated for both “Peak” and “Flank” regions (Figure 7B). Furthermore, the estimated 95% confidence intervals for “Peak” and “Flank” regions are not overlapping, indicating that the correlation of EnMuLV and SFFV mutational background is stronger under the “Peak” in recombinant frequency.

To test if the age of recombinants are consistent across different evolved viral populations, we repeated the above analysis with single post-passage viral population data. Like the aggregated data above, Spearman’s *Rho* and Kendall’s *Tau* correlations are significant between EnMuLV and SFFV mutational backgrounds for each independent post-passage line (Table 1, “All”) indicating that the recombinant region as a whole has been replicating in the viral population long enough to acquire a variant background similar to the homologous SFFV population of sequences. However, the pattern of higher correlations under the recombination “Peaks” compared to the “Flanks” are not consistent for all viral populations (Table 1). For both Spearman and Kendall’s correlations, only 3/6 comparisons show greater EnMuLV and SFFV mutational background correlation under the “Peak” in recombination, with only 2/6 Spearman and 1/6 Kendall comparisons showing significant differences (non-overlapping 95% CIs). However, for the comparisons showing greater correlation between mutational backgrounds for “Flank” positions (3/6), none of the differences are significant. The aggregate passage data show EnMuLV sequences at the “Peak” in recombination have been maintained in the viral population longer than the “Flanks” and thus have had more time to recombine with the SFFV population at large to acquire similar variant spectra. However, the patterns of individual passage lines are not as easily identified, making it impossible to determine if this is a general principle of SFFV populations experiencing recombinations with EnMuLVs.

**TABLE 1:** Spearman and Kendall rank correlations of EnMuLV background mutations.

Spearman’s rank correlation rho					
Host MHC Genotype	EnMuLV-like/SFFV Mutation Background Correlation				
	All	Peak	95% CI	Flank	95% CI
bb	0.54*	0.53*	0.46–0.60	0.54*	0.48–0.60
bb	0.37*	0.28*	0.19–0.36	0.42*	0.35–0.48
dd	0.50*	0.62*	**0.55–0.69**	0.45*	**0.38–0.53**
dd	0.46*	0.41*	0.32–0.49	0.49*	0.43–0.56
kk	0.53*	0.60*	0.52–0.67	0.50*	0.43–0.56
kk	0.33*	0.49*	**0.41–0.57**	0.33*	**0.25–0.39**

**Kendall’s rank correlation tau**					

**Host MHC Genotype**	**EnMuLV-like/SFFV Mutation Background Correlation**				
	
	**All**	**Peak**	**95% CI**	**Flank**	**95% CI**

bb	0.39*	0.38*	0.32–0.44	0.40*	0.35–0.45
bb	0.28*	0.21*	0.13–0.28	0.31*	0.26–0.37
dd	0.37*	0.46*	**0.41–0.53**	0.33*	**0.28–0.38**
dd	0.33*	0.29*	0.23–0.35	0.35*	0.31–0.41
kk	0.38*	0.44*	0.38–0.50	0.36*	0.31–0.41
kk	0.26*	0.35*	0.29 - 0.41	0.24*	0.19 - 0.30

*Bold values indicate non-overlapping 95% confidence intervals for Peak and Flank values.*

## Discussion

### Genetic Basis of FVC Adaptation to Host MHC

This is the first study to assess within-host evolution of FVC genomes and their interaction with endogenous retroviral elements using deep sequencing. Based on results of our experiments we were unable to detect a genetic signature of genotype-specific adaptation of FVC to host MHC via selective sweeps, despite strong phenotypic adaptation of virus to the MHC-host of passage (Figure 1; Kubinak et al., 2012); there were no fixed and few high frequency variants that emerged that were specific to a particular host MHC (Figure 2). However, we did detect signals of genotype-dependent viral adaptation when taking into account the full variant spectra of post-passage viral populations (i.e., greater overlap between replicate lines than between lines evolved in alternate host genotypes) (Figure 3). Interestingly, the degree of similarity of replicates is proportional to the known resistance of MHC haplotypes, with H-2^bb^ being most resistant to FVC and also leading to the most similar variant spectra and the opposite being true for the H-2^dd^ haplotype (Figure 3; Miyazawa et al., 2008). However, because of low sample size, caution should be taken when interpreting these data. Contrary to initial expectations, the above results suggest that combinations of low-frequency variants that uniquely arise in specific host genotypes may be responsible for the observed patterns of genotype-specific phenotypic adaptation.

Control of FVC is correlated with mouse CD4, CD8 and antibody responses (Hasenkrug and Chesebro, 1997; Myers and Hasenkrug, 2009). For instance, the H-2D^bb^ haplotype induces rapid CD4+ responses while H-2D^dd^ are much slower, leading to H-2D^dd^ being more susceptible to FVC induced disease (Miyazawa et al., 2008; Myers and Hasenkrug, 2009). Interestingly, there are few evolved Balb/c^bb^ SNVs detected within predicted H-2D^bb^ epitopes (Supplementary Table 2). The H-2D^bb^ haplotype being in a Balb/c background might alter its effectiveness at controlling FVC disease. For instance, the Ly49 NK receptors are non-MHC linked, but bind class I MHC molecules to cause either suppression or increases in NK cell immune activity (Millan et al., 2021). Additionally, Ly49 receptors have been identified on CD8+ T-cells involved in anti-viral responses (Shytikov et al., 2021). There are other non-MHC genotypes that interact with adaptive immune genes. For instance, Rfv3 (which encodes mouse APOBEC3) induces a swift virus neutralizing antibody response against FVC (Santiago et al., 2008). Thus, while not linked to MHC genes, its action is dependent on CD4 and B-cell activation. How these combinations of resistance/susceptibility alleles and MHC haplotypes affect the present study is unknown and warrants further research. However, if T-cell responses are weakened by Rfv3 or if Ly49/MHC interactions lead to altered NK immune pressures, the signatures of viral adaptation might not be in T-cell epitopes and thus hard to identify. This interpretation is consistent with evolved viral lines not showing enrichment of SNVs in T-cell epitopes (Supplementary Table 2).

The question of why genotype-specific adaptation does not leave a signature of selective sweeps (fixed mutations) is unclear, but several possibilities exist that could explain this outcome. First, the differential selective pressures due to MHC polymorphism in naive mice will act through the adaptive immune response, which takes about 6 days for T-cell responses to develop. With serial infections lasting 12 days, the virus population will only experience T-cell dependent selective pressure about half of the total time it is replicating and will experience a different selective pressure (e.g., the innate immune response) during the initial 6 days of infection (Hasenkrug and Chesebro, 1997; Myers and Hasenkrug, 2009). Second, and compounding the first issue, MHC-specific adaptations could reduce viral fitness during the initial phase of infection and thus, the abundance of MHC-specific variants may be actively selected against during this time. One test of this hypothesis could be to perform passages in mice which have been immunized against FVC or a subset of its proteins to decrease the time lag before selection via MHC-mediated adaptive immunity starts acting. Third, the responding host T-cell repertoire is in part determined by the frequency of viral alleles in a population. Thus, if a viral mutation escapes MHC recognition by changing an epitope so that it is no longer presented by MHC, different epitopes will likely become immunodominant in the next host (Walker and McMichael, 2012). The predicted end result of successive rounds of differential selection or shifting immunodominance against viral epitopes would be the emergence of a diverse variant spectra without representation of MHC-specific variants in the high-frequency pool. Given these possibilities, the low-frequency variant pool may contain the signature of genotype-specific adaptation, which is consistent with results from our MDS analysis (Figure 5). However it is at odds with our inability to identify overlap of evolved SNVs with predicted epitopes (Supplementary Table 2). This result should be interpreted cautiously, however, as epitope prediction *in silico* of FVC does not identify strong *in vivo* determined epitopes and might have limited utility.

Other mechanisms that could account for the lack of high-frequency host-genotype-specific viral variants could depend on viral population dynamics and diversity. First, genotype-specific selection may occur at the level of viral quasi-species (i.e., selection favoring specific combinations of variants rather than individual variants). Such cooperation among variant combinations has been demonstrated in other RNA viruses, such as the polio virus (Vignuzzi et al., 2006; Domingo et al., 2012). Our data supports this hypothesis by showing that, at least for MHC^bb^ and MHC^kk^ adapted virus populations, variant spectra are more similar when compared to virus populations derived from the same host genotype versus those adapted to different MHC genotypes (Figure 3). Since we did not sequence full-length genomes and do not have sufficient replicates to conclusively identify patterns of co-occurring mutations, future work will involve sequencing many more evolved viral lines with long-read technology like PacBio or Oxford Nanopore. Second, it is possible that the high multiplicity of infection (MOI) used in our serial passage model allows for accumulation of neutral or deleterious variants through complementation (more below). This could obscure patterns of mutation driven by host-mediated immune pressure by inflating the contribution of unselected variants. Alternatively, the accumulation of such variants could also contribute to quasi-species selection by providing immunological “cover” to adaptive variants from the immune response through distraction or T-cell anergy; where variants that alter immune epitopes and weaken T-cell binding can suppress activity of T-cells targeting the original viral genotypes (Sloan-Lancaster et al., 1993; Sadegh-Nasseri et al., 2010). This hypothesis has some precedence as FVC is known to induce tolerogenic states in responding T-cell populations (Takamura et al., 2010).

Several caveats must be kept in mind when interpreting the results described here. First, our sequencing approach leverages amplicon sequencing, thus it will miss genomic regions outside amplification products. These non-sequenced regions largely represent the long terminal repeats of both F-MuLV and SFFV, which could have mutations leading to increased viral fitness and virulence (such as regulatory mutations). Second, amplicon sequencing also runs the risk of missing viral haplotypes which have mutations within primer sites. This issue is greatest for the F-MuLV genome, which was amplified with three primer sets, however the overlapping nature of the amplification scheme allows for confirmation of primer site sequence. Sequencing results for F-MuLV show that there are no high frequency variants across the full length of the genome, indicating that the internal primer sequences likely do not have issues amplifying the majority of F-MuLV genotypes. Third, the experimental evolution regime did not bottleneck viral populations, potentially limiting the ability of the viral population to go through selective sweeps. This is expected to increase the amount of genetic variation. Thus, due to a high number of persisting genotypes, clonal interference could play a role in explaining the lack of evidence for selective sweeps in the viral populations (Muller, 1932).

### SFFV-EnMuLV Recombinants

Genetic exchange between exogenous and endogenous MuLVs and the loss of expression of host-encoded restriction factors can lead to novel viral genotypes and disease phenotypes. A well known case of this is in mice with *M. m. castaneus* and *M. m. domesticus* hybrid ancestry found near Lake Casitas in California. These mice have high MuLV viremia caused by loss of ecotropic restriction factors leading to unchecked replication of their EnMuLVs and recombination between ecotropic and polytropic EnMuLVs, which allows the ecotropic viruses to switch tropism. These new viral sequences lead to novel viral symptoms such as neuropathologies (Gardner et al., 1991). Common inbred strains of laboratory mice primarily have a *M. domesticus* ancestry, however several strains, such as AKR, also share some ancestry with *M. castaneus* and carry many copies of ecotropic MuLVs (Jenkins et al., 1982). These mice are prone to high levels of MuLV viremia; not unlike the hybrid mice of Lake Casitas. When ecotropic MuLVs are allowed to replicate in susceptible mice, like the *M. domesticus*-related inbred mouse lines, they can recombine with the defective polytropic EnMuLVs and cause severe disease. Thus, while SFFV-BC is a lab derived viral genome, recombinant viruses involving the resurrection of ERV sequences are potentially common in nature.

Through deep sequencing of post-passage SFFV populations, we found many common variants of SFFV which are the product of recombinations with EnMuLVs during passage. EnMuLV sequences are expressed in uninfected BALB/c mouse splenocytes, providing a ready-source of template for recombination with exogenous SFFV genomes. This genetic exchange between exogenous SFFV and endogenous EnMuLVs is associated with the rapid increase in viral titers and virulence previously observed for FVC adapted to specific MHC genotypes via serial passages (Kubinak et al., 2012). While some studies have identified EnMuLVs participating in recombination with SFFVs and what the end results are, they do not show to what extent the recombinations are ongoing and how they shape viral population structure. Two studies have identified endogenous polytropic MuLV sequences being packaged into F-MuLV virions as a common occurrence in NFS/N mice; happening as early as 1 day post-infection (Evans et al., 2009; Boi et al., 2016). Thus, endogenous sequences appear to be primed to not only recombine and create new viral genotypes, but also to continuously alter the SFFV viral population by repeated introgressions. The use of ultra-deep sequencing has allowed us to map the ongoing recombination landscape of replicating SFFV-BC and its recombinants derived from EnMuLVs. Sequencing has revealed that the SFFV-BC and EnMuLV-derived alleles show signs of linkage between positions of increased SFFV-BC and EnMuLV recombination (Figure 6). This implies that either there is selection to maintain separate SFFV-BC and EnMuLV haplotypes or that SFFV-EnMuLV recombinants are too young to have reached equilibrium through recombination with SFFV-BC. This fact, along with the observation of a peak in EnMuLV-derived variant frequencies, supports one of two non-mutually exclusive scenarios. First, a few EnMuLV to SFFV recombination events occurred and these were followed by positive selection, while secondary recombinations whittled away at the edges of the positively selected recombinant alleles. Second, that EnMuLVs are constantly introgressing into the replicating viral population and the peak in variant frequency simply reflects the EnMuLV introgression position probability.

Because ERVs are part of the host genome, their mutational loads reflect the host mutation rate, which are much lower than replicating retroviral populations (Aswad and Katzourakis, 2012). Once an ERV is “resurrected” into a replicating viral population, it will start accruing mutations at the rate of the viral population until it reaches a mutation/selection equilibrium. When looking at the mutational background of introgressed EnMuLV-like reads for aggregated data, it is clear that EnMuLV sequences have similar mutational loads as SFFV-like reads (Figure 7A). This indicates that replicating EnMuLV-like sequences have reached mutation/selection balance or are close to it, which suggests that the recombinant sequences are maintained and actively replicated within the viral population. Therefore, it is likely that the SFFV population was invaded by resurrected ERV sequences early and that they are not replaced by new recombinants fast enough to balance the mutational rate. It is unclear, however, at what rate replicating SFFV would reach mutation/selection balance from a clonal population, so placing a lower bound on when the EnMuLV sequences invaded the SFFV population is not currently possible. Sequencing virus from a time-series of mice infected with clonal virus could be used to calibrate the mutational load to resurrection-age calculations.

These data lead to a paradox: why do the EnMuLV-like sequences look as if they are being selected for, with a peak in variant frequency (Figures 2B, 7A), and show signs of being in the viral population long enough to reach mutation/selection balance (Figure 7A), yet they never reach fixation? If recombinant alleles had even a modest selective advantage (∼1%) and arise early in passage [as early as 1 day post infection (Evans et al., 2009) and at least by round 2 of 10 passages in this experimental setup (Supplementary Figure 5)], theory would predict they should reach fixation in the additional ∼100 days of viral replication. One possible explanation is that recombinants do not represent more fit variants, but rather accumulate as byproducts of recurring recombination that cannot be purged from the viral population; a mechanism analogous to defective-interfering (DI) particles. DIs accumulate rapidly because of deletions in their genomes that reduce nucleotide polymerization time (Garcia-Arriaza et al., 2004). The accumulation of these viruses can begin to negatively influence the fitness of non-defective related viruses at some frequency threshold (Huang, 1973). When MOI is high, as expected in this study, SFFV genotypes unable to induce erythroproliferation may accumulate because their deficiency can be complemented by inocula-type erythroproliferative SFFV.

In this scenario, these recombinants are detrimental to FVC replication, yet the high rate of introgression makes it impossible for the viral population to purge them. Thus, the observed peak in recombinant sequences would represent not the most-fit, but rather the least deleterious part of the recombinant, with the flanking regions being selected against more strongly. This would resolve the paradox of recombinants not going to fixation, because they would be dependent on replicating exogenous SFFV to activate the EpoR and cause cell proliferation. If this is true, damping the evolution of viral fitness with the constant introduction of defective genomes into replicating populations could have major implications for the adaptation of retroviruses. This would imply that retention, expression, and recombination of EnMuLVs can be a host adaptation to infections with related retroviruses.

Recombination with EnMuLVs could impact FVC evolution in several other ways. Clearly, EnMuLVs provide the raw genetic material for the generation of novel phenotypes, as was the case for the evolution of SFFV itself (Friend, 1957; Hasenkrug and Chesebro, 1997). However, if recombinants are not as fit as SFFV-BC-like genotypes, the recombinants could reduce SFFV’s ability to fix new adaptive mutations through clonal interference, slowing its adaptation to host genotypes. Likewise, the recombinants could act as a static immune target, being less variable than SFFV genotypes. This isn’t without merit because immune reactions targeting ERV peptides have been discovered in humans and ERV-targeting antibodies and T-cell responses are positively correlated with HIV control (SenGupta et al., 2011; de Mulder et al., 2017). Furthermore, in the ongoing genome invasion of koalas with Koala retrovirus (KoRV), koalas possessing certain endogenous KoRV subtypes show altered disease progression in response to exogenous KoRV infection compared to those that lack these endogenous KoRV (Quigley et al., 2019), and koalas vaccinated against an endogenous KoRV-A subtype are protected against exogenous KoRV (Olagoke et al., 2020).

Future work could test the hypothesis that SFFV/EnMuLV recombinants are less fit and only viable with high MOI by infecting mice with serially diluted post-passage FVC stocks. If the SFFV-EnMuLV:SFFV-BC ratios become higher with increasing MOI, it would suggest that MOI, rather than selection is the primary driver of recombinant frequencies. The fate of recombinant sequences could be tested with artificial SFFV/EnMuLV populations harboring non-natural silent mutations (as markers) to aid in determining the half-life of viral sequences relative to the introgression of naturally occurring EnMuLV sequences. Likewise, titering EnMuLV-like sequences in artificial populations could also test whether ERV sequences are dampening viral fitness and virulence. Finally, the immune-mediated protective nature of ERV recombinants could be tested by immunizing mice against peptides encoded by these EnMuLVs and performing experimental infections to test viral fitness and virulence. These future efforts will be critical for understanding how ERV introgression influences retroviral fitness and virulence, which as highlighted above, is an ecologically relevant question that needs to be addressed.

## Conclusion

Our initial goal of discovering the genetic basis for the observed adaptation of FVC to specific host MHC genotypes revealed there were few unique high-frequency mutations specific to viral lines adapted to an MHC. These data rejected the leading hypothesis that selective sweeps of host-genotype-specific adaptations were responsible for the observed phenotypic patterns (Figure 1). We provide numerous mechanisms that could explain how causal variants might remain at low frequencies. Accordingly, when looking at full variant spectra (which includes low frequency variants) a potential signature of host genotype-specific adaptation is observed (Figure 3). Through this work we have also demonstrated that the genome of SFFV readily recombines with related endogenous retroviruses during serial passage in BALB/c mice. Interestingly, these recombinants do not reach fixation after ten 12-day passages, but show signatures of selection centering on a 12 bp insertion. This indicates that the recombinants are maintained through several generations of viral replication and are packaged into viral particles. Our demonstration of rampant recombination between endogenous and exogenous MuLVs during active infection suggests that this could be a powerful evolutionary force. Whether it is recombinational happenstance or is being favored by selection because it provides an advantage to the virus or host, remain open questions. Approximately 10% of mammalian genomes are retroviral sequences inserted during past infections and this huge store of genetic material could be used by virus or host to their own advantage (Kozak, 2015; Katzourakis and Aswad, 2016; Johnson, 2019). In this study, the introgression of ERV sequences of EnMuLV origin into viral genomes were the highest frequency variants observed, though they never reached fixation. Thus, we cannot exclude the possibility that these recombinants are of low fitness and that their high frequency is due to high rates of recombination between ERVs and infectious viral genomes. This is an intriguing point because it suggests that endogenous retroviruses may be co-opted by hosts to degrade the genomic integrity (and consequently pathogenicity) of exogenous viral populations through recombination.

## Materials and Methods

### Viral Passage Lines

Previously, using serial passage, FVC was adapted to specific host MHC genotypes, resulting in trade-offs in viral fitness and virulence when alternative MHC genotypes were infected with these evolved viral lines (Kubinak et al., 2012). Briefly, 10 rounds of 12-day passages were performed in laboratory mice. To increase the probability of passage success, the passage rounds included two mice at each round. At the end of each round, spleens from each mouse was homogenized and the supernatants were pooled. This pooled inocula was used to infect mice intraperitoneally for the following round. After the last passage round, virus pools were used to infect individual mice. For this study, one mouse infected with its respective post-passage viral stock was used for sequencing viral pools. For example, spleens from two MHC^bb^ mice (one infected with bb replicate one passage stock and another infected with bb replicate two passage stock) were used for sequencing. The same strategy was used for sequencing dd and kk viral lines derived from passage in MHC^dd^ and MHC^kk^ mice, respectively. This lead to a total of 120 mice being used for passages (6 lines ^∗^ 2 mice ^∗^ 10 rounds) and 6 mice for sequencing (1 per line). For sequencing, spleens from infected mice were homogenized in 1X PBS (equal organ weight:PBS volume) using a tephlon bit spun at approximately 300 rpm. 100 μL homogenates were used in DNeasy Blood and Tissue DNA Extraction Kits (Qiagen) following the recommended animal tissue protocol. DNA extractions from these six individual mice were used for generation of all Illumina sequencing data described in this study. For passage details see Kubinak et al. (2012). For details of the Non-recombinant viral stock used in section “**SFFV Recombination-Specific PCR**,” see *Virulent FVC Stock* from Cornwall et al. (2021).

### Viral Amplification and Sequencing

To detect variants selected for during serial passage of FVC, complete viral sequences were obtained using an Illumina next-gen approach. Viral sequences were amplified using overlapping PCR amplicons. Primer sets were based on the F-MuLV reference sequence X02794.1 and SFFV reference sequences K00021.1 and V01552 (Supplementary Table 1). Amplification was performed with ThermoFisher Phusion Taq using manufacturer recommended reagent and cycling conditions [with the exception of added DMSO (0.04%) and an annealing temperature of 65°C, extension time of 45 s for F-MuLV fragments and 90 s for the SFFV fragment]. Amplicons were gel purified with the QIAquick Gel Extraction Kit (Qiagen) and then cleaned in an additional cleanup step using the DNA Clean and Concentration Kit (Zymogen), both according to manufacturer’s protocols. Amplicons were aliquoted in 25 μL at a concentration of 15 ng per μL. These were sent to the Wisconsin National Primate Research Center to be pooled in equal concentrations. Libraries were constructed with Illumina’s transposase based Nextera reagents following manufacturer’s protocol. Sequencing reads were generated on Illumina’s MiSeq platform with paired-end 250 bp chemistry. A sequencing depth of at least 10,000X was generated for each sample. Raw sequencing data generated for this manuscript are available in NCBI’s SRA under BioProject PRJNA736962.

### SFFV *de novo* Assembly

Illumina sequencing reads from SFFV-BC were randomly down-sampled with the program SeqTk (version 1.0-r31) to obtain approximately 100,000 fastq entries (Shen et al., 2016). Raw.fastq files were filtered and trimmed with Trimmomatic using a 5′ and 3′ quality cutoff of 13 along with a 4 bp sliding window quality filter of 20 (Bolger et al., 2014). This .fastq file was used as input for the SPAdes *de novo* assembler version 2.4.0 (Bankevich et al., 2012) with a kmer value of 121. The output was automatically sorted by scaffold length. The longest scaffold was used in a BLAST search against the NR nucleotide database with NCBI’s web interface (Zhang et al., 2000; Morgulis et al., 2008).

### Variant Detection Pipeline

Raw.fastq files were filtered and trimmed with Trimmomatic using a 5′ and 3′ quality cutoff of 13 along with a 4 bp sliding window quality filter of 20 (Bolger et al., 2014); reads less than 30 bp were removed. Filtered read-pairs were mapped with Bowtie2 (version 2.3.0) to the pre-passage pathogen reference genomes (Langmead and Salzberg, 2013). Samtools was used to sort and remove duplicate reads (Li et al., 2009). To account for base mis-calls due to insertions and deletions, an indel realignment was performed with GATK3 (McKenna et al., 2010). Finally, the pile-up and variant calling was performed with the program SNVer, which is designed to be used with high-ploidy samples like viral populations, with a ploidy setting of 200, minimum mapping quality of 10, and a p-value cutoff of 0.01. The BASH script used to implement this pipeline is found at https://github.com/EarlyEvol/FVC_manuscript/FVC_Variant_detection.sh.

### MDS and Variant Spectra Comparisons

Variant files (.vcf) from the alignment and variant calling protocol were formatted and concatenated into three column files, one file each for F-MuLV and SFFV SNVs. These files were passed through a custom R script to create the MDS and variant spectra distance figures. Briefly, concatenated variant data for all samples was formatted into a data frame suitable for distance matrix calculation with the aid of “setDT” from the package *data.table* (Dowle and Srinivasan, 2021). The function “metaMDS*”* from the *vegan* package with method “Canberra” was used to compute the pairwise multivariate distance between samples and generate the MDS values used in Figures 5A,B (Oksanen et al., 2020)^1^. The Canberra method has the advantage of deemphasizing zero values, which are common in the comparisons due to many SNV frequencies being near or below the limit of detection. Distance matrices were used to generate pairwise “Same” vs. “Diff” distance comparisons (Figures 3C,D).

### Epitope Overlap With Evolved SNVS

Immunogenic epitopes of F-MuLV were predicted with the web tool at http://tools.iedb.org/main/tcell/ (accessed 7/2021). Epitopes of MHC class I alleles (H-2-Dd, H-2-Kd, H-2-Db, H-2-Kb) were predicted with method NetMHCpan EL 4.1 with option “all lengths.” MHC class II epitopes of alleles (H-2-IAd, H-2-IAb) were predicted with method “IEDB recommended 2.22” and allowed to span lengths 12–18. Additionally, *in vivo* derived T-helper inducing epitope sequences were obtained from Messer et al. (2014). A python script was written to identify epitope locations on the F-MuLV genome, filter predicted epitopes by percentile MHC binding (>99.5% and 98 for MHCI and MHCII epitopes respectively), and calculate number of non-synonymous SNVs within 3 nucleotides for each passage line. Results are presented in Supplementary Table 2. See https://github.com/EarlyEvol/FVC_manuscript/SNV_epitope_overlap.py for full script. A full list of epitopes is available in Supplementary File “H2-D_K_IA_FMuLV_epitopes.csv”.

### SFFV Recombination-Specific PCR

Primers were designed to distinguish SFFV-BC sequence from the SFFV/ERV recombinant detected from deep sequencing reads. The first forward primer corresponds to the ERV sequence, which shows a 12 bp insertion relative to the SFFV reference (SFFV_recombF), with the other forward primer corresponding to the SFFV reference sequence at the same position (SFFV_non_recombF). The reverse primer used with both of these forward primers corresponds to inoculum sequence approximately 1000 bp downstream (SFFV_non_recombR). DNA was isolated with the protocol described in Viral Passage Lines. The presence of the SFFV recombination is identified by a positive PCR product when SFFV_recombF and SFFV_non_recombR are used, while a PCR product from SFFV_non_recombF and SFFV_non_recombR was used as a positive control for SFFV reference sequence. Amplification was performed with ThermoFisher Phusion Taq using manufacturer recommended reagent and cycling conditions [with the exception of added DMSO (0.04%), an extension time of 15 s, and annealing temperature of 65°C]. Primer sequences can be found in Supplementary Table 1.

### RNA Alignments

BALB/c splenocyte RNA reads were obtained from NCBI’s short read archive (Accessions: ERR216358, ERR216374, ERR216375, ERR216376, ERR216377). The .fastq files were concatenated and reads trimmed with Trimmomatic using 5′ and 3′ quality cutoff of 13 along with a 4bp sliding window quality filter of 20 (Bolger et al., 2014). Alignment to SFFV-BC was performed with Bowtie2 version 2.3.0 with discordant reads pairs being discarded and using minimum and maximum insert length of 0 and 2000 bp, respectively (Langmead and Salzberg, 2013). Samtools was used to sort, remove duplicates, and index alignment files (Li et al., 2009). To do local indel realignments, GATK3 was used with default parameters. Finally, variants were called using SNVer with an expected ploidy of 200, minimum mapping quality of 10, and a *p*-value cutoff of 0.01. For details of analysis pipeline see https://github.com/EarlyEvol/FVC_manuscript/FVC_Variant_detection.sh.

### Recombination Hotspots

Recombination between SFFV-BC and EnMuLV genomes were inferred from passaged virus sequencing reads to identify recombination hotspots and potential selection for the resulting products. Illumina reads used for post-passage variant detection were aligned to a consensus reference sequence derived from an alignment of SFFV-BC and a representative EnMuLV (GRCm39: chr12 69546811–69555877), where each nucleotide difference was replaced with an “N.” A custom script^2^ was then used to classify each read as (1) containing only SFFV-BC alleles, (2) containing only ERV alleles, or (3) containing both. For reads that have both SFFV-BC and ERV SNVs, recombinations were inferred if they meet at least one of two criteria. The first criterion only called a read recombinant if the SNVs changed from SFFV-BC to ERV once in either orientation, i.e., the read was one half SFFV-BC and one half ERV. To capture multiple recombination events per read, the second criterion called all allele changes recombinations as long as there were two consecutive SFFV-BC or ERV SNVs on each side of the inferred recombination regardless of the alleles on the rest of the read.

### Mutational Background Analysis

SAM file format alignments of post-passage virus Illumina reads aligned to the SFFV-BC reference sequence were passed through a custom python script to identify read SNVs as either EnMuLV or novel mutations^3^. Briefly, EnMuLV SNVs were identified from uninfected BALB/c splenocyte RNA-seq (from “RNA Alignments” above) and filtered for those present at ≥ 2% of the viral population. After realignment around indels, reads were then interrogated to identify base identity at EnMuLV SNV positions. These identities were used to classify regions between EnMuLV-like SNVs as originating from EnMuLV recombinations. Total numbers of EnMuLV-like SNVs were used to infer the total EnMuLV frequency at any given site, with values for positions between the EnMuLV SNVs being inferred by simple linear interpolation. The rest of read positions were interrogated for differences from the SFFV-BC reference sequence. Any identified differences were characterized as novel mutations. Novel mutations were then classified as originating on an EnMuLV background if both flanking sites were identified as EnMuLV-like. If not, the mutation was classified as originating on the SFFV background. Results were then output as a.csv file containing per-read mutational backgrounds for SFFV-BC and EnMuLV-like reads standardized by their inferred respective coverages.

### Statistical Analysis and Data Visualization

Single sample *t*-tests were used to test if variant spectra from hosts with the same MHC genotype were more similar to each other than they were to spectra of viral populations from different MHC genotypes. To do this, the built-in base R function t.test was used. Independent statistical comparisons were performed on F-MuLV and SFFV variant spectra. For the first MHC^bb^ sample, a list of bb vs. dd, bb vs. dd.rep, bb vs. kk and bb vs. kk.rep Canberra distances were used as the reference distribution and bb vs. bb.rep as the single distance to be tested. These comparisons were repeated for the bb.rep sample (i.e., bb.rep vs. dd, bb.rep vs. dd.rep, bb.rep vs. kk and bb.rep vs. kk.rep as the reference distribution), and likewise for the other two host MHC genotypes. When aggregating all distances of virus from different host genotypes, the data are consistent with a normal distribution, tested by the base function shapiro.test (F-MuLV *p*-value:0.65, SFFV *p*-value:0.66). However, when doing single comparisons, we lack the power to assess the normality of data with only four values to compare. Even so, of the 12 tests (6 for F-MuLV and 6 for SFFV), 11 have data consistent with a normal distribution. Only when comparing the variant spectra of kk.rep to all others is the assumption of normality violated (Shapiro–Wilks test; *p*-value: 0.03). For this comparison, the kk.rep vs. kk Canberra distance is less than all of the distances of kk.rep to viruses from different MHC genotypes, supporting the conclusion of the *t*-test. For mutational background analysis, correlation statistics were generated with the R packages *stats*^4^ for Spearman and Kendall correlation tests and *NSM3* and *RVAideMemoire* for Kendall and Spearman correlation confidence interval modeling, respectively (Schneider et al., 2020; Hervé, 2021). Data were visualized using ggplot2 (Wickham, 2016). Likewise, plots of inferred recombinational hotspots (Figure 6) and MDS and distance plots (Figure 5) were generated with the R package ggplot2.

## Data Availability Statement

Data presented within this manuscript are available at NCBI’s SRA under Bioproject PRJNA736962 (Raw reads) and GenBank under accession MZ614724 (*de novo* assembled SFFV sequence).

## Author Contributions

EM, WP, and JK conceived of this study. EM, DC, and DS generated the data. EM performed the program development and data analysis. EM and WP wrote the manuscript with editing and revisions provided by JK, DC, and DS. All the authors contributed to the article and approved the submitted version.

## Conflict of Interest

The authors declare that the research was conducted in the absence of any commercial or financial relationships that could be construed as a potential conflict of interest.

## Publisher’s Note

All claims expressed in this article are solely those of the authors and do not necessarily represent those of their affiliated organizations, or those of the publisher, the editors and the reviewers. Any product that may be evaluated in this article, or claim that may be made by its manufacturer, is not guaranteed or endorsed by the publisher.

## Footnotes

1
https://cran.r-project.org/web/packages/vegan/

2
https://github.com/EarlyEvol/FVC_manuscript/blob/master/SFFV_v_ERV_recomb.pyand

3
https://github.com/EarlyEvol/FVC_manuscript/blob/master/age_of_recomb.RNAseq.manuscript.py

4
https://rdocumentation.org/packages/stats/versions/3.6.2
