# Supplementary material for: Deep Sequencing of MHC-Adapted Viral Lines Reveals Complex Recombinational Exchanges With Endogenous Retroviruses Leading to High-Frequency Variants
Source: Front Genet. 2021 Aug 27;12:716623. doi: 10.3389/fgene.2021.716623 (PMC8430262; doi:10.3389/fgene.2021.716623)
Supplement: Supplementary file 2 [file Data_Sheet_2.pdf]

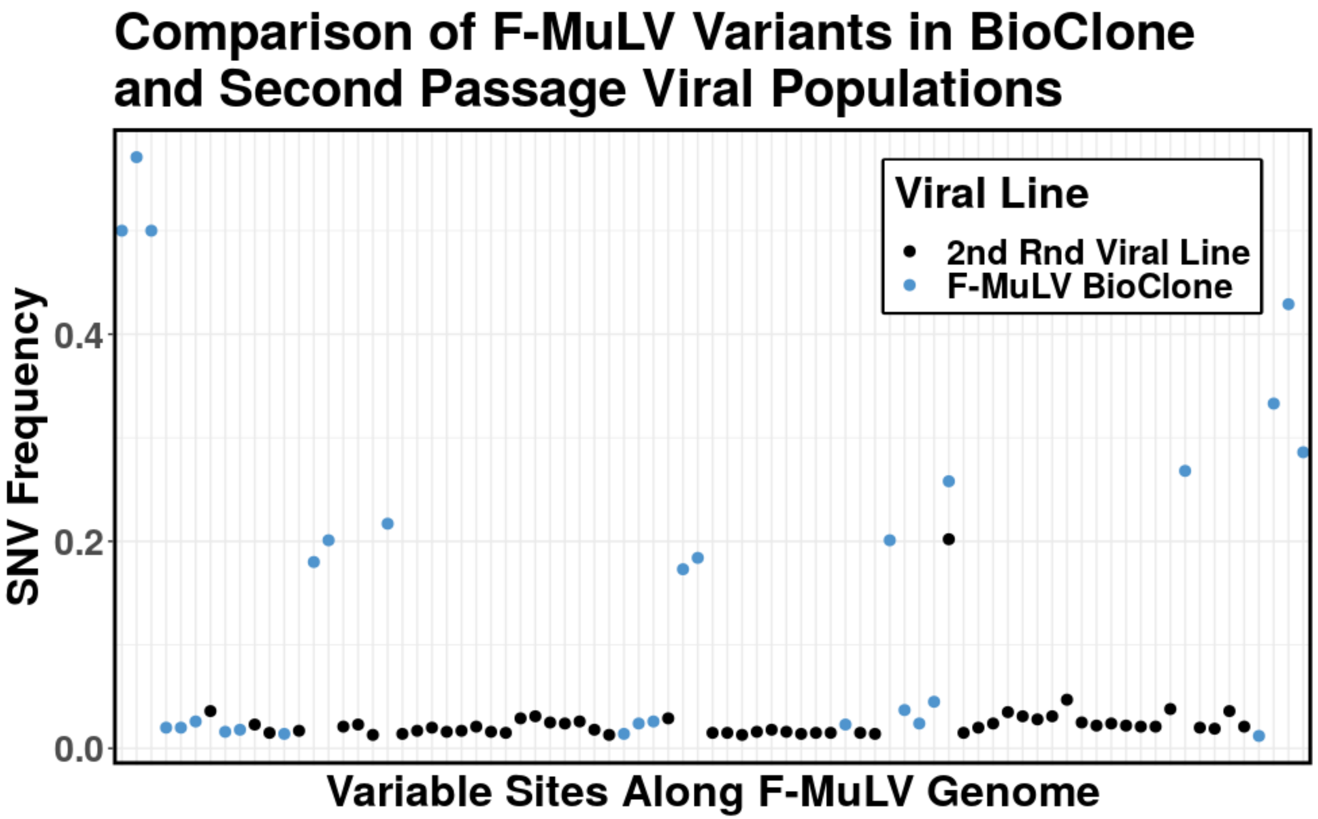

Supplementary Figure 1 F-MuLV variants detected in cell culture virus stock "BioClone" are absent from virus sequenced after 2 rounds of passage in DBA mice. Each variable genomic position is given a column. Zero columns have both black and blue points, meaning that there are no shared variants between the F-MuLV BioClone (inocula) population and an F-MuLV viral population after 2 rounds of passage in mice. This illustrates that BioClone variants are 1) likely caused by drift or cell culture adaptation and 2) viral gains in fitness and virulence from passage 3-10 are not due to these variants being purged from the viral population.

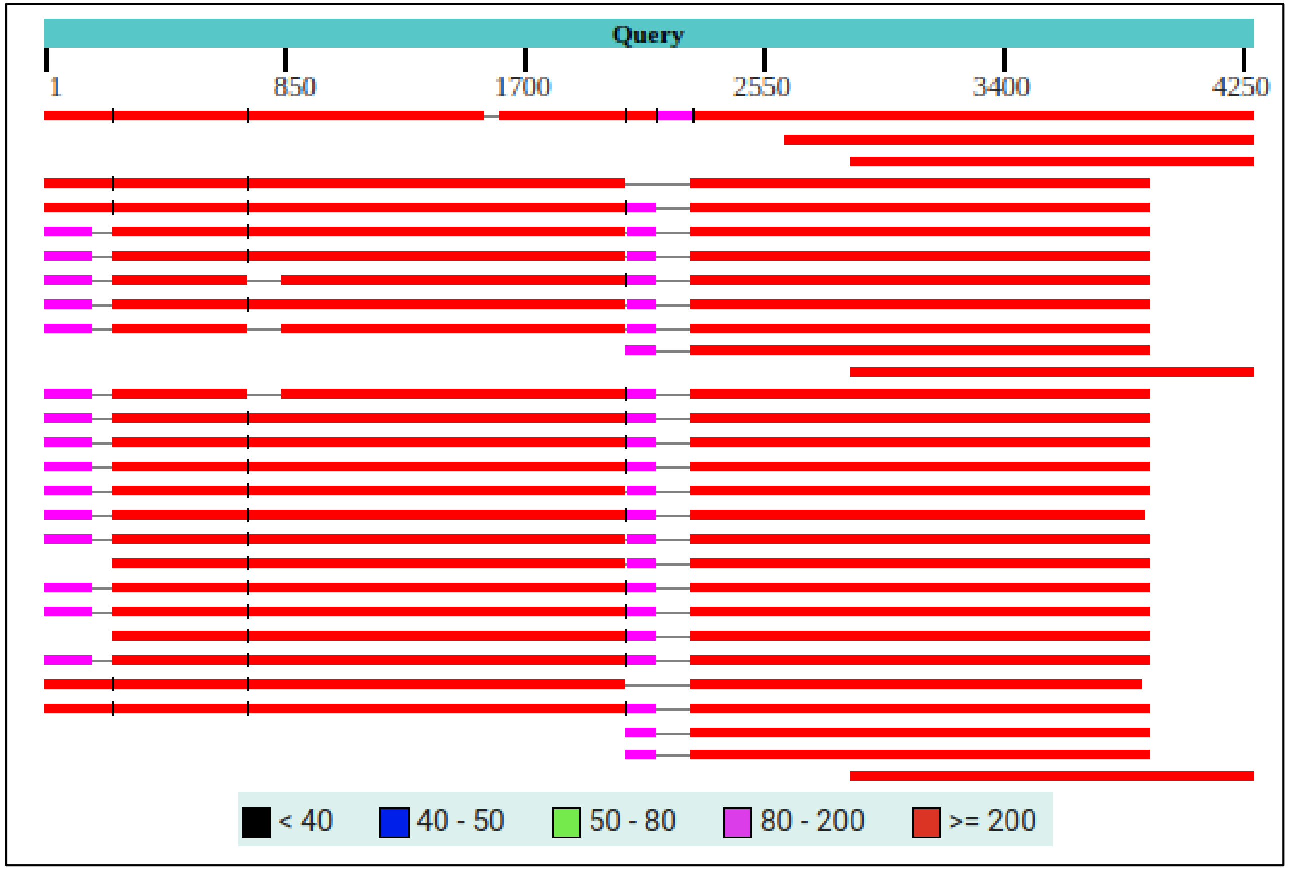

Supplementary Figure 2 Visualization of alignments generated by BLASTn of the *de novo* generated SFFV-BC sequence as a query against the NCBI NR/NT database. The alignment position relative to the query is shown at the top of the figure. Colored bars depict alignments of sequences from NCBI's nr/nt database with each color indicating the alignment score calculated by NCBI's megablast BLASTn algorithm. Grey lines connecting bars represent gaps in alignments due to extra sequence in the query, while vertical black bars show gaps due to extra sequence in the blast hit.

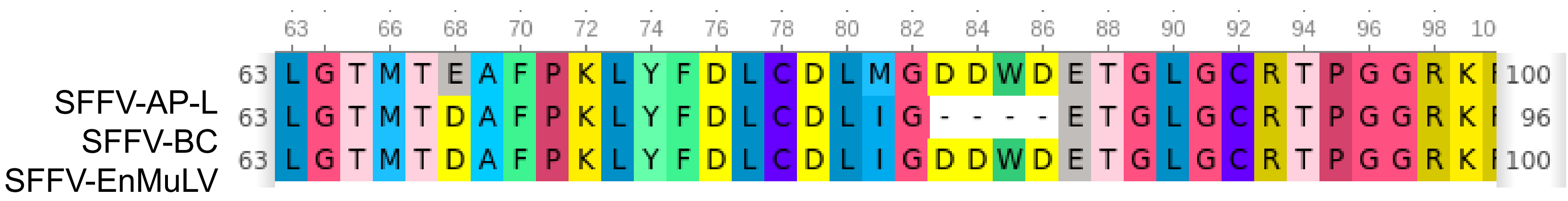

Supplementary Figure 3. Comparison of the inferred protein products of the *de novo* assembled SFFV genome (SFFV-BC), a published, high virulence SFFV (SFFV-AP-L), and the detected post-passage recombinant (SFFV-PR). The incula SFFV genome (SFFV-BC) shows a 12 base pair deletion relative to the high virulence strain. The recombination of SFFV-BC with endogenous MuLVs to create SFFV-EnMuLV introduces an apparent 12bp insertion. This makes the recombinant peptide sequence similar to that of the high virulence SFFV-AP-L with a "DDWD" motif that was lacking in SFFV-BC.

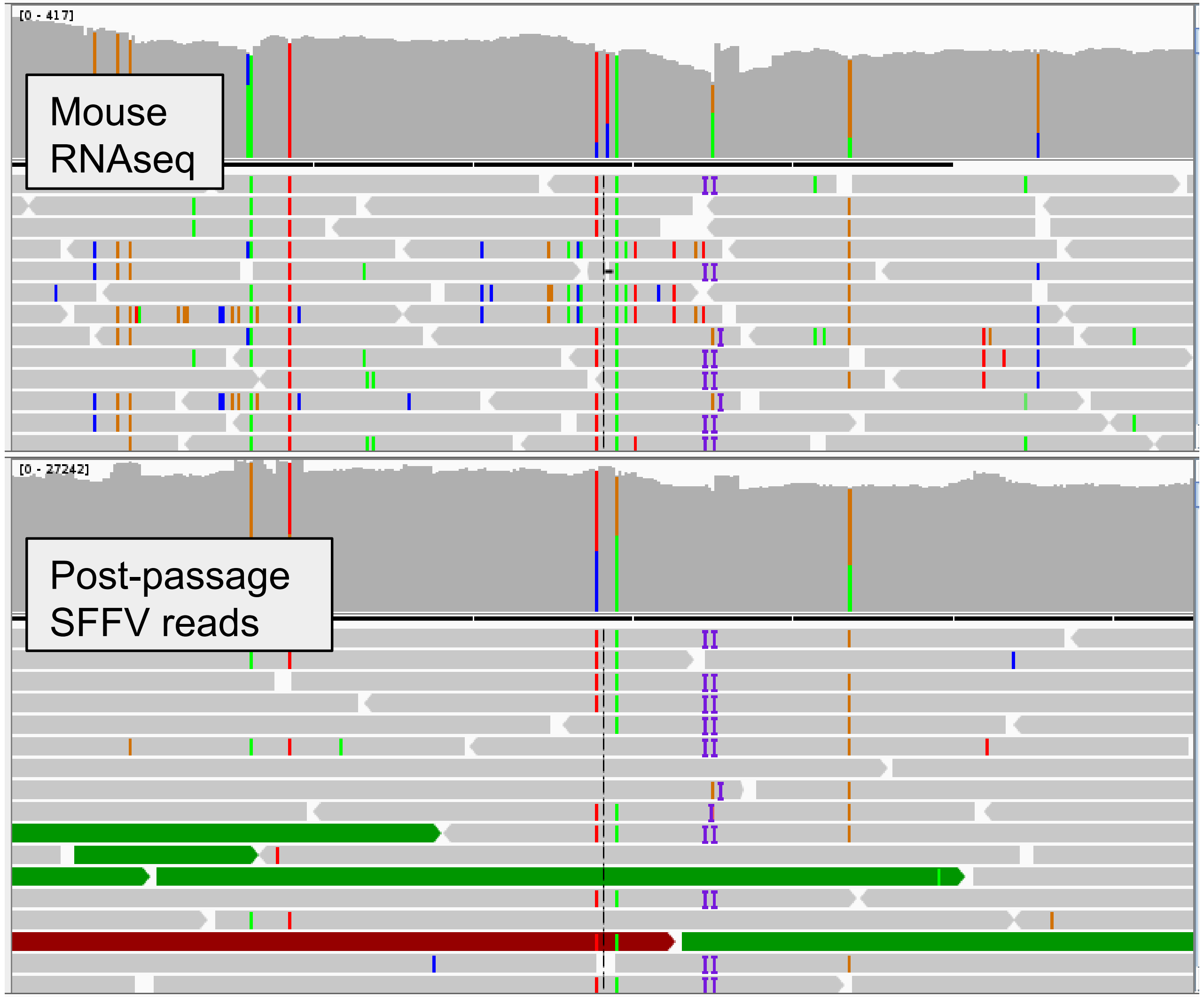

Supplementary Figure 4. Comparison of alignments of illumina RNA-seq reads from uninfected mouse splenocytes and post-passage FVC reads to SFFV-BC (position 2950 to 3320). SNPs are shown as colored bars in read pile-up and coverage map, with percent allele identity shown by hight of colors in coverage map. Insertions and deletions are shown as purple brackets and black dashes, respectively. Values in the top left of each panel show the coverage units. Reads depicted as green or maroon are from read pairs which aligned discordently. Comparing the top and bottom panels shows that many SNVs and indels found in aligned RNA-seq reads from uninfected mice are also found in post-passage SFFV reads. Furthermore, many of the mutations are linked on the same reads, indicating they did not independently arise, but rather have a simillar origin.

supplementary

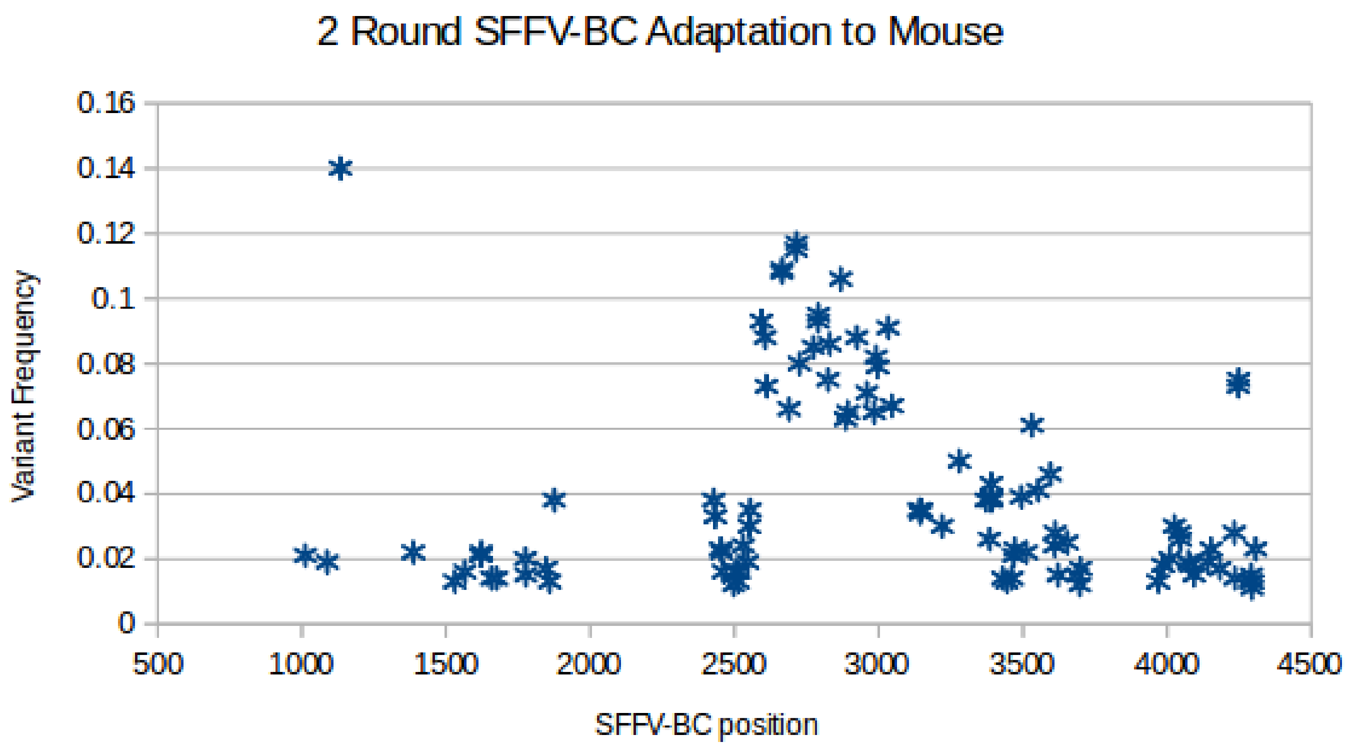

Supplementary Figure 5 Variant frequency of post passage SFFV-BC after 2 rounds of mouse adaptation is shown. Sequencing reads were generated on an Illumina MiSeq instrument with libraries constructed using a Nextera prep of post passage SFFV whole genome amplicons. Variants were detected with the bioinformatic Pipeline in additional methods **Alignment and Variant Detection**. The high frequency variants in the plot mirror EnMuLV associated variants discussed throughout this manuscript.

supplementary

Supplementary Table 1: Sequences of primers used for NGS sequencing and recombinant PCR

Primer Name	Template	sequence
Seq_FMuLV_Fragment_1_F	F-MuLV	GTCCATTGTCCCGTGTCTTTGATTG
Seq_FMuLV_Fragment_1_R	F-MuLV	GTGGAGTCTCAGGCAGAAGAAAGC
Seq_F-MuLV_Fragment_2_F	F-MuLV	GGT AAG GTC ACC CAC TCT TTC CTC
Seq_F-MuLV_Fragment_2_R	F-MuLV	CGATTCTGCTGGTGGCTCAATC
Seq_F-MulV_Fragment_3_F	F-MuLV	GTCAAGCAAGGGACTAGAGTTCG
Seq_F-MulV_Fragment_3_R	F-MuLV	CTG TTT GGC CCA GCG TAT CTG
Seq_SFFV_Fragment_1_F	SFFV-BC	GTT TTG TCT CCG TCT CTG TCT TTG TG
Seq_SFFV_Fragment_1_R	SFFV-BC	GCGTTACAGCGGCATCAGG
recomb_SFFV	EnMuLV	ATTAGGGGACGACTGGGATG
non-recomb_SFFV	SFFV-BC	GCGATTTAATAGGAGAGACTGGAC
SFFV_non-recombR	SFFV-BC	GATTAAGCACGGTGGCAGAT

Supplementary Table 2: Overlap of non-synonymous SNVs detected from NGS reads and T-cell epitopes. Comparisons of both computationally predicted and empirically identified epitopes are shown for each passage line (2 per MHC haplotype). Cells highlighted in blue show matching host-of-passage and host genotype origin of the T-cell epitope. For MHCb and MHCd haplotypes, 162 and 98 non-redundant epitopes were predicted (supplementary file “Filtered_epitopes”). The full list of predicted epitopes and their binding percentile predictions can be found in supplementary file “H2-D_K_IA_FMuLV_epitopes”.

	Epitope Haplotype	Viral Host Genotype	Number of SNVs			Cumulative Variant Frequency		
			Epitope Associated SNVs	All SNVs	Fraction Associated with Epitope	Epitope Associated SNVs	All SNVs	Fraction Associated with Epitope
H-2 D, K and IA*	MHC bb	Balb/cbb	4	45	0.09	0.06	1.23	0.04
		Balb/cbb	3	19	0.16	0.08	0.51	0.16
		Balb/cdd	5	26	0.19	0.13	0.54	0.24
		Balb/cdd	4	42	0.10	0.12	1.28	0.09
		Balb/ckk	2	16	0.13	0.11	0.47	0.24
		Balb/ckk	8	59	0.14	0.13	2.96	0.04
	MHC dd	Balb/cbb	3	45	0.07	0.04	1.23	0.03
		Balb/cbb	4	19	0.21	0.13	0.51	0.25
		Balb/cdd	2	26	0.08	0.10	0.54	0.19
		Balb/cdd	4	42	0.10	0.16	1.28	0.13
		Balb/ckk	4	16	0.25	0.13	0.47	0.29
		Balb/ckk	1	59	0.02	0.01	2.96	0.00
Empirical CD4**	MHC bb	Balb/cbb	2	45	0.04	0.03	1.226	0.02
		Balb/cbb	1	19	0.05	0.03	0.513	0.06
		Balb/cdd	0	26	0.00	0.00	0.54	0.00
		Balb/cdd	2	42	0.05	0.024	1.282	0.02
		Balb/ckk	0	16	0.00	0.00	0.468	0.00
		Balb/ckk	2	59	0.03	0.023	2.958	0.01

*Epitope prediction from http://tools.iedb.org/main/tcell/

**Empirical epitopes reported in (Messer, Lavender, and Hasenkrug 2014)
